# Ten simple rules for implementing electronic lab notebooks (ELNs)

**DOI:** 10.1371/journal.pcbi.1012170

**Published:** 2024-06-20

**Authors:** Justine Vandendorpe, Beatrix Adam, Jeanne Wilbrandt, Birte Lindstädt, Konrad U. Förstner

**Affiliations:** 1 ZB MED—Information Centre for Life Sciences, Cologne, Germany; 2 Leibniz Institute on Aging–Fritz Lipmann Institute, Jena, Germany; 3 TH Köln–University of Applied Sciences, Cologne, Germany; Carnegie Mellon University, UNITED STATES

## Introduction

An electronic lab notebook (ELN) is a software tool for documenting laboratory experiments, research data, and processes. ELNs are set to replace paper lab notebooks as part of the ongoing digital transformation [1].

ELNs offer many benefits over physical lab notebooks. First, ELNs are connected to a networked digital environment [2] through their import and export functions and seamless interfaces to other programmes. ELNs can therefore play a central role in an institution’s Research Data Management (RDM) strategy (see definition in S1 Text). Second, ELNs enable researchers to collaborate [3], whether within their own group or with others, through a common medium. Third, ELNs make an important contribution to Good Research Practice (GRP) [4] by facilitating the tracking, tracing, and documentation of research processes and results through time. ELNs also prevent data loss and guarantee data security. Finally, ELNs support the FAIR data principles [5], for example, through the assignment of metadata, tags, and persistent identifiers (PIDs).

In order to successfully implement an ELN in your institution, we recommend that you involve staff from different areas (i.e., researchers, professors, lab managers and assistants, IT experts, librarians, and staff council (see definition in S1 Text)) at all stages of product selection and evaluation to achieve the greatest possible consensus.

The following 10 rules offer guidance on implementing an ELN in your institution, bearing in mind that each institution may require a solution tailored to its specific needs. We also discuss the benefits of open-source ELNs and the importance of standardisation.

## Rule 1: Gather information

There are many resources available to help you familiarise yourself with the ELNs currently available.

For example, ZB MED provides an ELN Guide [6] designed to support research infrastructure teams and researchers in selecting an ELN. The ELN Guide also includes helpful references. Together with the University and State Library Darmstadt, ZB MED also created the ELN Finder [7], an interactive tool for filtering ELNs based on 40 criteria. The Harvard Longwood Medical Area Research Data Management Working Group also provides an ELN Comparison Matrix [8], while guides are offered by the ELN providers Labfolder [9] and SciNote [10], the University of Cambridge [11] and uncountable [12].

You can also use the working groups, networks, mailing lists, and best-practice examples listed in Table 1 to help you select and use an ELN. Joining a working group is a good way to exchange information and find out what solutions other users have developed, such as discipline-specific adaptations.

**Table 1:** Communities and resources focusing on ELNs.

Name	Type	Range	URL
ELB.nrw	Working group	Germany	https://wiki.hhu.de/display/ELB/ELB.nrw+Startseite
National Research Data Infrastructure working group on ELNs	Working group	Germany	https://www.nfdi.de/section-infra/?lang=en
Ouvrir la science—Cahiers de laboratoire électroniques	Working group	France	https://www.ouvrirlascience.fr/cahiers-de-laboratoire-electroniques/
Research Data Alliance (RDA)—Research Data Architectures in Research Institutions IG	Network	International	https://www.rd-alliance.org/groups/research-data-architectures-research-institutions-ig
The ELN Consortium	Consortium	International	https://github.com/TheELNConsortium
Elabnotebook	Mailing list	Germany	https://listserv.gwdg.de/mailman/listinfo/elabnotebook
German Research Network	Mailing list	Germany	eln@listserv.dfn.de
UK Education and Research communities	Mailing list	UK	https://www.jiscmail.ac.uk/cgi-bin/webadmin?A0=RESEARCHNOTEBOOKS
NFDI4Chem, Chemotion	Best-practice example	Germany	[6]
University Medicine Göttingen, RSpace	Best-practice example	Germany	[6]
ETH Zurich, openBIS	Best-practice example	Switzerland	[6]
University of Edinburgh, RSpace	Best-practice example	UK	[6]

## Rule 2: Define selection criteria

To select ELNs for testing, we suggest that you define selection criteria that reflect the needs of your institution and lab(s). By comparing your list of criteria with the features offered by available ELNs, you can systematically reduce the number of ELNs to test. You can also filter the results of the ELN Finder [7] based on your criteria to see which tool(s) it suggests.

This section covers what we consider to be the most important criteria; a full list of criteria can be found in the appendix (S2 Text) (e.g., templates, task assignment, rights and inventory management, security level of your data, budget, possibilities for collaborative development and expansion).

The first thing to consider is the research discipline behind the ELN. There are discipline-specific ELNs (e.g., Chemotion for chemistry, eLabJournal for molecular biology, PsychNotebook for psychology), but they may not meet the needs of your subdiscipline, especially if it is niche. One way to address this is to consider ELNs used by other institutions within your network/community.

Second, choose between a proprietary (e.g., eLabJournal, Labfolder, RSPace) and open-source ELN (e.g., Chemotion, openBIS, SciNote). Open-source ELNs offer the benefits of digital sovereignty [4], though they can be time-consuming to run and maintain and may require the purchase of server hardware unless managed by a service provider. Open-source ELNs do not have a fixed, vendor-defined set of features, and you will be dealing with a community of developers instead of a single vendor. Some open-source software communities are very active and have multiple contributors, while others are not. For example, as of 22 February 2024, eLabFTW has 41 contributors who have consistently maintained the code for the past 12 years, with new issues opened on 21 February 2024 and new commits to the code base on 17 February 2024 [13]. Finally, open-source ELNs may lack support services and could complicate the task of meeting regulatory requirements. Demo instances of various open-source ELNs are provided by the German National Research Data Infrastructure for Chemistry (NFDI4Chem) [14].

Proprietary ELNs offer the benefits of being developed by vendors who regularly update their ELN and provide training and support services. On the other hand, proprietary ELNs are expensive, and the vendor may go out of business or raise their prices. Additionally, proprietary ELNs use proprietary formats, which may increase the risk of vendor lock-in (i.e., where users become dependent on the ELN and are unable to move their data to another ELN without significant migration costs [15]). To mitigate this risk, you should make sure your chosen ELN allows you to export and archive all your data and/or the entire ELN in open formats [4] (e.g.,.xml,.csv,.png,.svg). There is currently no universally accepted open exchange format, but there are standardisation initiatives (e.g., Standards4ELN [16] with the development of a minimal metadata set and a transfer software package, e.g., using RO-Crate; The ELN Consortium [17] with its ELN File Format [18]).

You will also need to choose between cloud-hosted Software as a Service (SaaS) and locally hosted, on-premises solutions. SaaS gives you the ability to use the ELN without the need for your own infrastructure [1], but it is expensive [4], and data control and security remain in the hands of the ELN provider (check your funder/institutional policy to see if you are allowed to use such solutions) [1]. On-premises solutions give you full control over data and security protocols, but require dedicated IT staff (e.g., for administration). That also means you may need a server to run your ELN and a server to store your data.

Finally, you should consider the performance and stability of both the ELN and the company/developer community [1] by answering the following questions:

- How long has the company/developer community existed?
- Does it have enough capital investment to withstand crises?
- Can it provide references?
- How many people does it employ? Does it have a professional development team?
- Do its employees have a research background and/or extensive experience in the lab?
- Does it state in its terms and conditions that users will have access to their data if the company/developer community goes under or is sold? [1]

## Rule 3: Test for usability in practice

**Fig 1:**
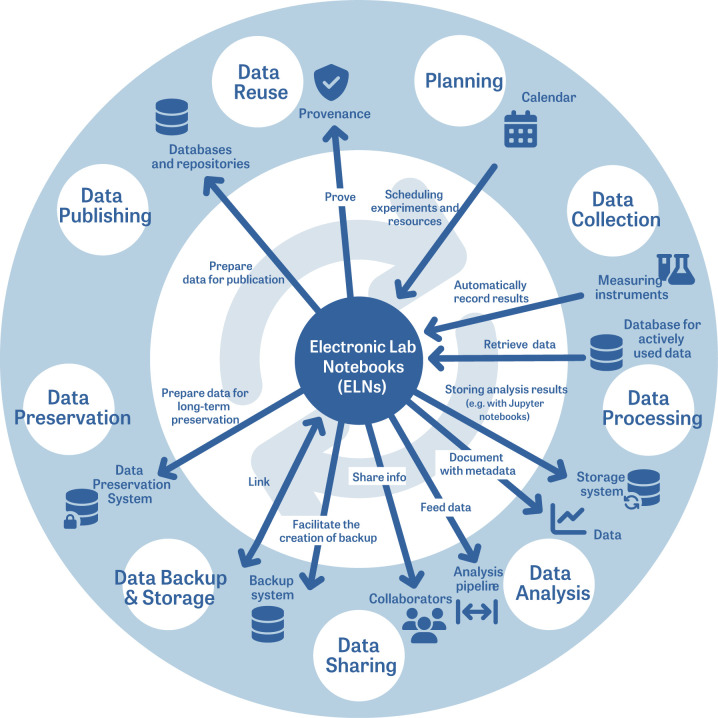
The role an ELN can play in the research data lifecycle.

Usability testing is a technique used to evaluate ELNs [19] by conducting extensive tests that are as close to reality as possible. This testing phase should help you to determine the suitability of ELNs in an everyday working environment [1], to assess where your risk lies in choosing a particular ELN and to figure out where the ELN fits in the research data life cycle (see definition in S1 Text) (Fig 1).

There are a number of approaches that can be taken to testing: for example, identifying individual laboratories willing to pilot the use of an ELN and act as a source of feedback. Additionally or alternatively, we recommend testing the ELN in parallel with the use of a physical lab notebook to (1) prevent data loss if the tested ELN is not selected; (2) document the usability testing phase to justify the selection process afterwards; (3) determine the extent to which real workflows can be implemented in the ELN; and (4) determine whether the ELN can actually produce the required documentation with additional benefits (e.g., making data searchable and reusable and making research findings reproducible) compared to physical lab notebooks. If the ELN does not provide these additional benefits, there might be other issues to consider.

For this usability testing phase, we suggest that you first set up a test team of researchers and lab assistants. Ideally, this test team would meet weekly with the IT coordinators and data stewards (see definition in S1 Text). Next, select the workflows and prepare a test questionnaire (see ours in S3 Text). It may also be helpful to create a matrix of criteria and ELNs to tick off what has already been tested. Finally, you need to determine the order in which the ELNs will be tested.

We recommend taking an initial look at a wide range of ELNs and then preselecting 2 or 3 tools to test over a period of 3 to 6 months, depending on how long it takes to gain sufficient knowledge of the tools’ suitability. Almost all companies will provide a trial version of their ELN on request, and NFDI4Chem provides demo instances of various open-source ELNs [14].

## Rule 4: Handle sensitive data appropriately

If you intend to collect sensitive data with the ELN, you should involve your institution’s data protection officer (see definition in S1 Text) in the implementation process to ensure compliance with your institution’s data protection regulations and the General Data Protection Regulation (GDPR) (see definition in S1 Text). These regulations may restrict the physical location and transfer of data, excluding the use of cloud-hosted ELNs [1,4]. You can also contact your RDM support team to learn more about anonymisation and pseudonymisation services, as well as your organisation’s existing policies and procedures for handling sensitive data and how an ELN might fit into this picture. Ideally, this should be done at the stage of writing your Data Management Plan (DMP) (see definition in S1 Text) [20]. It is important to note that privacy issues are not addressed by the ELN itself, but rather by how you set it up and store your data.

## Rule 5: Introduce the ELN

The introduction of an ELN begins with the actual installation or establishment of the service. For a proprietary ELN, the first step is to obtain the necessary licence. For a smooth introduction, the next step—for both proprietary and open-source ELNs—is to ensure a signed agreement is in place between management and staff regarding staff monitoring. Monitoring staff with an ELN is technically possible, but the pros and cons need to be carefully considered. You should also involve your institution’s data protection officer and staff council in this process in order to ensure compliance with institutional regulations and the GDPR.

Introduction of the software itself starts with ensuring that all technical requirements are met such as a stable wireless internet connection, suitable end devices with up-to-date hardware and software [4]. Suitable end devices need to be stationed in appropriate locations, and there should be enough of them; if there are not enough, funding may be needed to purchase them. Suitable end devices would be computers or tablets that can be handled with gloves or that come with a tablet pen (which should be used with gloves on). Depending on the biosafety level of your lab, these tablets may need to be specially encased. Both computers and tablets should be protected from liquids. By allowing access to the ELN via appropriate devices that remain in the lab, an ELN can prevent the easy contamination of both physical and electronic lab notebooks [4].

You then need to establish and execute a rollout plan, which could include the following steps: setting up the software, administering the system, troubleshooting, distributing the ELN to labs, providing support, and applying updates as and when required. Users and disseminators (e.g., RDM trainers, data stewards) need to be trained (e.g., hands-on, face-to-face training) and support services need to be set up (e.g., wiki, hotline, FAQ and key contacts). The final step in introducing the tool is to establish an offboarding procedure to ensure that outgoing user data is properly archived [4]. For this step to be successful, roles and responsibilities need to be defined: it should be clear who will create the rollout plan, who will provide training, and who will promote the ELN and introduce it to different parts of the institution.

## Rule 6: Collect feedback

Feedback from researchers and lab assistants can be gathered in stages. First, monitor usage of the ELN to determine whether it is well accepted by the labs. If a group is not using the ELN much, identify the reason and try to support the group. Second, visit individual labs to get detailed feedback. The focus here is on the interaction between the research staff and data stewards responsible for the ELN. The aim is to identify where support (e.g., hands-on, face-to-face training) is needed and how processes (e.g., documentation, ergonomics) could be improved. Third, establish a systematic feedback collection process based on a questionnaire, an interview, or group discussions. This will help you to decide which new developments and/or product modifications should be commissioned. Even at times when you are not collecting feedback, keep the communication channels open. Ongoing support is key to successful implementation [4].

## Rule 7: Provide efficient documentation

ELNs offer features and functions that can provide significant time savings and knowledge transfer in day-to-day lab work. These typically include the ability to create templates (e.g., for protocols, standard operating procedures (SOPs), and workflows), automatically record instrument results [21], retrieve data from databases for active data, capture unstructured data, and annotate raw data (e.g., with metadata, tags) without having to switch between different media formats. Tips and tricks for using an ELN as efficiently as possible can be found in documentation (e.g., eLabJournal’s Best Practice Use Guide [22]), training from your institution or the ELN provider (e.g., training sessions offered by eLabFTW [23]), video tutorials (e.g., ZB MED’s videos on eLabFTW and Labolder [24]), and community meetings (e.g., bi-monthly for eLabFTW [25]). Ideally, your lab should also have its own operating procedures for best use of the ELN [3], which could be shared with other labs using the same ELN. Other resources that could be developed and shared by your institution include case stories with the user community of a specific ELN and the creation of mailing lists or similar channels for your institutional users to share information about ELN developments.

## Rule 8: Integrate the ELN in your RDM strategy

As a comprehensive data documentation tool, the ELN can play a central role in your institution’s RDM strategy. ELNs allow templates to be created for data collection and facilitate the enrichment of data with metadata. ELNs also allow data to be captured as early as possible and fed directly into an analysis pipeline [4]. The assignment of PIDs can also be incorporated into ELN systems. ELNs facilitate backup, publication, and archiving [21] by providing the means to prepare data for these steps and by linking to reference management software databases (e.g., RSpace links to the University of Edinburgh’s DataStore [2], other ELNs link to DataVerse and PubMed [3]), repositories (e.g., RSpace links to the University of Edinburgh’s DataShare [2], Chemotion links to Chemotion Repository and can link to other repositories [7]), and digital preservation systems (see definition in S1 Text; e.g., RSpace links to the University of Edinburgh’s DataVault [2]) (please check with your local RDM staff what are the rules for storing data at your institution). This can help you to meet the requirements of funders and publishers to make your data reusable by helping you store, reference, publish, and archive your data for the long term. Finally, ELNs can prove provenance [3] through audit trails and documentation of both data generation and equipment used.

To facilitate the integration of the ELN into your RDM strategy, there are a number of requirements to consider. Once you have a server to run your ELN and a server to store your data, you will need policies for backing up data and preserving it for at least 10 years.

It is also possible to make the ELN interact with commonly used software, programming languages (e.g., Python [26]), data management platforms (iRods [27]), and laboratory equipment (e.g., FLUICS label printers [28]), e.g., through application programming interfaces (APIs). Finally, it is important to enable links to files internal to the ELN and external to the ELN, as well as file-sharing services (e.g., Academic Torrents, B2DROP, Open Science Framework (OSF), DataLad, FAIRDOM-SEEK) and repositories (see examples above).

Another way to facilitate the integration of the ELN into your RDM strategy is to use an open exchange format (see Rule 2 for more details) that allows you to export your data from the ELN and use it in other systems.

## Rule 9: Build a community

To share your experiences, you can build a community within your institution around the ELN you have implemented. This community can focus on user support, which is essential for successful implementation. It can be built around peer-to-peer support and address issues such as accessibility, instructions on how to use the ELN, and problem solving (see *Rule 7: Provide efficient documentation* for ideas on how to share information about ELNs within your institution, e.g., with mailing lists).

You can also build or join a community around your ELN outside your institution. Consider maintaining contact with the ELN provider or the open-source community developing the ELN to communicate your needs for further development (e.g., by joining the bi-monthly community meetings organised by eLabFTW [25]). Also consider joining a network with other institutions using the same ELN to share experiences, resources (e.g., training videos for open-source ELNs, test questionnaires, needs analysis survey, templates), and solutions (e.g., storage connectivity, integration into your RDM strategy).

## Rule 10: Contribute to the ELN

Open-source ELNs allow you both to observe the developments of other users and to make your own contributions. This makes it possible to extend the tool to meet your (discipline-specific) needs and to share these developments in a documented way (e.g., with eLabFTW [29]).

An important way to contribute is by improving the core source code. This involves actively participating in the development of the fundamental infrastructure of the ELN, implementing new features, improving existing features, and optimising performance.

You can also contribute by developing and improving plugins/extensions (you can create your own plugins with eLabJournal, Labfolder, and openBIS.). These add-ons extend the capabilities of the ELN and help to meet the specific needs of subcommunities (e.g., eLabJournal has a CloneAssist extension [7] to visualise DNA and plasmids and apply various molecular cloning techniques [30], Labfolder has a Figshare extension [7] to transfer scientific data directly from the ELN to the scientific archive [31], and openBIS has a JupyterHub extension [7] to launch Jupyter notebooks directly from the openBIS interface [30]). By creating or enhancing these additional components, you can help diversify and enrich the ELN’s feature set.

Other contributions (e.g., for non-coders) include testing the developer version, reporting bugs with a detailed description of the problem, and writing documentation.

## Conclusions

ELN implementation should be carried out using a top-down approach: the decision should come from the top, as should the resources for software, support and staff training, the formalisation of ELN use in a policy, and best practices for data and documentation management. To be sustainable, however, ELN use must be driven by personal conviction. Implementing and using an ELN requires time, ongoing support, and training, but will be rewarded with increased efficiency, collaboration, and trust in your work.

## Supporting information

S1 Text Glossary of terms specific to research data management. (DOCX)

S2 Text Criteria for selecting an electronic laboratory notebook (ELNs) for testing. (DOCX)

S3 Text Questionnaire to test the usability of ELNs. (DOCX)
