# Supplementary material for: Ten simple rules for implementing electronic lab notebooks (ELNs)
Source: PLoS Comput Biol. 2024 Jun 20;20(6):e1012170. doi: 10.1371/journal.pcbi.1012170 (PMC11189195; doi:10.1371/journal.pcbi.1012170)
Supplement: S1 Text — (DOCX) [file pcbi.1012170.s001.docx]

Ten Simple Rules for Implementing Electronic Lab Notebooks (ELNs)

Justine Vandendorpe (ORCID: 0000-0002-9421-8582)^1^, Beatrix Adam (ORCID: 0000-0002-8431-6613)^1^, Jeanne Wilbrandt (ORCID: 0000-0002-0363-3837)^2^, Birte Lindstädt (ORCID: 0000-0002-8251-1597)^1^, Konrad U. Förstner (ORCID: 0000-0002-1481-2996)^1, 3^

^1^ ZB MED - Information Centre for Life Sciences, Cologne, Germany

^2^ Leibniz Institute on Aging – Fritz Lipmann Institute, Jena, Germany

^3^ TH Köln – University of Applied Sciences, Cologne, Germany

*foerstner@zbmed.de

# S1 Appendix Glossary

- **Data Management Plan (DMP):** formal and living document that defines responsibilities and provides guidance. It describes data and data management during the project and measures for archiving and making data and research results available, usable and understandable after the project has ended
- **Data protection officer:** person who oversees the application of and compliance with regulations designed to protect important information from corruption, compromise or loss within an organisation [1, 2].
- **Data steward:** expert in the preparation and management of data, including data selection, storage, preservation, annotation, provenance and other metadata maintenance, and dissemination [3].
- **Digital preservation system:** tool for managing the activities (e.g., planning, risk management, preservation activities) needed to ensure continued access to and independent understanding of digital materials by a designated community for as long as it is needed [4, 5].
- **General Data Protection Regulation (GDPR):** regulation “on the protection of natural persons with regard to the processing of personal data and on the free movement of such data” [6].
- **Research data life cycle:** model that illustrates the steps of research data management and describes how data should ideally flow through a research project to ensure successful data curation and preservation.
- **Research Data Management (RDM) strategy:** series of measures that need to be taken during a research project in order to (1) obtain high-quality data (whether produced or reused), (2) make the data available and usable over the long-term and (3) make research findings reproducible beyond the research project.
- **Staff council:** body of elected representatives of employees and civil servants [7].

# Reference

1. Data protection officer. 2023 Oct 17 [cited 01 March 2024]. In: Data Management Wiki [Internet]. Herveld: DAMA-NL 2023 - . [about 1 screen]. Available from: <https://datamanagement.wiki/role/data_protection_officer>.
2. Crocetti P, Peterson S, Hefner K. What is data protection and why is it important? 2021 Feb [cited 01 March 2024]. In: TechTarget [Internet]. Newton: TechTarget 2024 - . [about 8 screens]. Available from: <https://www.techtarget.com/searchdatabackup/definition/data-protection>.
3. European Commission. Directorate General for Research and Innovation., EOSC Executive Board. Digital skills for FAIR and Open Science: report from the EOSC Executive Board Skills and Training Working Group. [Internet]. LU: Publications Office; 2021. Available from: <https://data.europa.eu/doi/10.2777/59065>.
4. What is digital preservation? [cited 01 March 2024]. In: Digital Preservation Coalition [Internet]. Glasgow: Digital Preservation Coalition 2024 - . [about 1 screen]. Available from: <https://www.dpconline.org/digipres/what-is-digipres>.
5. CCSDS. Recommendation for Space Data System Practices REFERENCE MODEL FOR AN OPEN ARCHIVAL INFORMATION SYSTEM (OAIS) RECOMMENDED PRACTICE. 2012 [cited 01 March 2024]. In: PLOS Blogs [Internet]. Available from: <https://public.ccsds.org/pubs/650x0m2.pdf>.
6. European Union. EUR-Lex - 02016R0679-20160504 - EN - EUR-Lex. 2016 [cited 01 March 2024]. In: Europa.eu [Internet]. Luxembourg: Eur-Lex. Available from: <https://eur-lex.europa.eu/legal-content/EN/TXT/?uri=CELEX%3A02016R0679-20160504>.
7. Duties and Responsibilities. [cited 01 March 2024]. In: University of Tübingen [Internet]. Tübingen: University of Tübingen 2024 - . [about 1 screen]. Available from: <https://uni-tuebingen.de/en/facilities/staff-representatives-advisory-services/staff-council/staff-council-duties-and-responsibilities/>.
