# Supplementary material for: Ten simple rules for implementing electronic lab notebooks (ELNs)
Source: PLoS Comput Biol. 2024 Jun 20;20(6):e1012170. doi: 10.1371/journal.pcbi.1012170 (PMC11189195; doi:10.1371/journal.pcbi.1012170)
Supplement: S2 Text — (DOCX) [file pcbi.1012170.s002.docx]

Ten Simple Rules for Implementing Electronic Lab Notebooks (ELNs)

Justine Vandendorpe (ORCID: 0000-0002-9421-8582)^1^, Beatrix Adam (ORCID: 0000-0002-8431-6613)^1^, Jeanne Wilbrandt (ORCID: 0000-0002-0363-3837)^2^, Birte Lindstädt (ORCID: 0000-0002-8251-1597)^1^, Konrad U. Förstner (ORCID: 0000-0002-1481-2996)^1, 3^

^1^ ZB MED - Information Centre for Life Sciences, Cologne, Germany

^2^ Leibniz Institute on Aging – Fritz Lipmann Institute, Jena, Germany

^3^ TH Köln – University of Applied Sciences, Cologne, Germany

*foerstner@zbmed.de

# S2 Appendix. Selection criteria

Three ELN systems are defined below, and criteria to consider when selecting an ELN are listed for each system.

### Basic ELNs

Basic ELNs are tools that are used as ELNs but that were not originally developed for this purpose (e.g., Word, Evernote, Dropbox) [1]. They allow for traditional text entries that can be searched and made available via the cloud. They also allow files to be attached, viewed, annotated and searched.

### Specialised ELNs

Specialised ELNs are tools that allow unstructured data entry and offer an extensive range of functionalities (e.g., eLabJournal, eLabFTW, Labfolder) [1]. In addition to all the features of basic ELNs, they also have the ability to capture freehand and chemical drawings [2] and offer subject-specific features/editors and templates. Moreover, they enable task assignment to multiple individuals, complex rights management, basic inventory management (i.e. allowing the quantity and location of samples and reagents to be managed), and extensions/APIs for customisation. Last but not least, they comply with the FDA 21 CFR Part 11 (USA) [3] and the EU Annex 11 [4]. These regulations require full audit trails, electronic signatures on completed records, witnessing and freezing, and measures to prevent records being deleted by their author.

### High-end ELNs

High-end ELNs are tools that come as a module of a comprehensive laboratory management system [1] (e.g., Hivebench, Limsophy). In addition to all the features of specialised ELNs, they include a Laboratory Information Management System (LIMS) that allows complete tracking of samples and reagents through all experiments. They are also directly linked to laboratory equipment and can automatically deliver raw data and metadata from this equipment. Finally, high-end ELNs provide workflows and allow data mining and analysis of raw data within the ELN.

### Further criteria to be considered

Other criteria to consider when selecting an ELN include those discussed earlier in this paper. You will need to choose between a proprietary and an open-source ELN; if you choose a proprietary ELN, it will need to provide an appropriate exit strategy/full export capabilities, including of the entire ELN. You will also need to choose between a cloud-hosted Software as a Service (SaaS) or a locally hosted, on-premises solution, and you should consider the performance and stability of both the ELN and the company/developer community (see Rule 2). You should also consider usability (i.e. clarity, intuitive operation, easy to navigate menus, drag and drop) (see Rule 3).

Other criteria include your lab's established practices and preferences, the security level of your data, your budget [5], whether you want a browser-based solution, the transparency and flexibility of the company/developer community, the possibilities for collaborative development and expansion, and the location of the headquarters.

# Reference

1. Dirnagl U, Przesdzing I. A pocket guide to electronic laboratory notebooks in the academic life sciences [Internet]. Vol. 5, F1000Research. F1000 Research Ltd; 2016. p. 2. Available from: <http://dx.doi.org/10.12688/f1000research.7628.1>.
2. Harvard Longwood Medical Area Research Data Management Working Group. Electronic Lab Notebook Comparison Matrix [Internet]. Zenodo; 2021. Available from: <https://zenodo.org/record/4723753>.
3. Part 11, Electronic Records; Electronic Signatures - Scope and Application. 2018 Aug 24 [cited 04 Dec 2023]. In: U.S. Food & Drug Administration [Internet]. Silver Spring: FDA - . [about 8 screens]. Available from: <https://www.fda.gov/regulatory-information/search-fda-guidance-documents/part-11-electronic-records-electronic-signatures-scope-and-application>.
4. European Commission, Enterprise and Industry. EU GMP Annex 11: Computerised Systems. 2011 Jun 30 [cited 04 Dec 2023]. In: ECA Academy [Internet]. Mannheim: ECA Foundation 2023 - . [5 pages]. Available from: <https://www.gmp-compliance.org/guidelines/gmp-guideline/eu-gmp-annex-11-computerised-systems>.
5. Electronic Lab Notebooks. 2023 [cited 17 November 2023]. In: Longwood Medical Area Research Data Management Working Group (LMA RDMWG) [Internet]. Boston: Harvard College 2023 - . [about 2 screens]. Available from: <https://datamanagement.hms.harvard.edu/collect-analyze/electronic-lab-notebooks>.
