# Supplementary material for: Ten simple rules for implementing electronic lab notebooks (ELNs)
Source: PLoS Comput Biol. 2024 Jun 20;20(6):e1012170. doi: 10.1371/journal.pcbi.1012170 (PMC11189195; doi:10.1371/journal.pcbi.1012170)
Supplement: S3 Text — (DOCX) [file pcbi.1012170.s003.docx]

Ten Simple Rules for Implementing Electronic Lab Notebooks (ELNs)

Justine Vandendorpe (ORCID: 0000-0002-9421-8582)^1^, Beatrix Adam (ORCID: 0000-0002-8431-6613)^1^, Jeanne Wilbrandt (ORCID: 0000-0002-0363-3837)^2^, Birte Lindstädt (ORCID: 0000-0002-8251-1597)^1^, Konrad U. Förstner (ORCID: 0000-0002-1481-2996)^1, 3^

^1^ ZB MED - Information Centre for Life Sciences, Cologne, Germany

^2^ Leibniz Institute on Aging – Fritz Lipmann Institute, Jena, Germany

^3^ TH Köln – University of Applied Sciences, Cologne, Germany

*foerstner@zbmed.de

# S3 Appendix. Test questionnaire

This questionnaire is broadly based on the ZB MED ELN Guide (pp 45 to 49) [6].

### Lab requirements

Does the ELN allow you to:

- Import and process special scientific formats?
- Export and process special scientific formats?
- Create your own templates (with drop-down menus to choose from a specified vocabulary)?
- Import templates or offer preconfigured templates? If so, which formats are supported?
- Share templates
  - With members of your own research group?
  - With external members who have a licence for the same ELN?
  - With external members who have no ELN licence?
- Link to files outside the ELN?
- Work concurrently on the same documents as several other collaborators?

Does the ELN offer:

- The special data entry and processing aids you need (e.g., scientific calculator, formula editor, dictation devices, digital pen, animal module, antibodies module, plasmids module, strains and cells module)?
- Sample management and the possibility to create material databases within the ELN?
- Standard interfaces (e.g., connection to a Laboratory Information Management System (LIMS) or other software)?
- Collaboration functions (e.g., individual users and groups, role management)?

### Usability

- Is the ELN easy to navigate?
- How easily can you access a folder you have uploaded and are now looking for?
- Are the options self-explanatory?
- Does the ELN allow you to insert images and tables?
- Does the ELN allow you to use any text formatting you want?
- Does the ELN allow for freehand drawing?
- Does the ELN organise data in a way that meets your needs?
- Are you confident the ELN will be easy to explain to senior lab members who are used to physical lab notebooks?
- Are you confident the ELN will be easy to explain to new lab members?

### Good Research Practice (GRP)

Does the ELN allow you to:

- Search for keywords and experiments within the ELN using free text search, advanced search, database queries and special search queries (e.g., searches for chemical formulas and reactions)?
- Ensure evidentiary value (e.g., audit trail, electronic signature and time stamp)?
- Easily export backups? If yes, in which formats?
- Easily export the entire ELN (e.g., for archiving purposes, or to migrate it to another ELN)?

Does the ELN offer:

- Documentation and traceability in line with GSP?
- Compliance with special regulatory requirements (e.g., GxP, ISO, FERPA, HIPAA)?
- Access (at least “read only”) for a minimum of ten years?
- (In-built) archiving / backups?

### Connection to Research Data Management (RDM) system

Does the ELN allow you to:

- Assign persistent identifiers (e.g., Digital Object Identifiers (DOIs))?
- Reference an open experiment or sample (e.g., with a Persistent Identifier (PID)), even if the folder structure changes in the future?

Does the ELN offer:

- Support for assigning metadata (e.g., input masks)?
- Automatic metadata harvesting (e.g., extraction from log files, export from Excel spreadsheets)?
- Integration of controlled vocabularies?
- A connection to repositories, publishing platforms, file sharing services?
- A connection to digital preservation systems?

### IT and data security

Does the ELN allow you to:

- Protect sensitive data within the ELN?
- Store data locally at the institute or in the institute’s own cloud?
- Run it on all operating systems (OS)?

Does the ELN offer:

- Application Programming Interfaces (APIs)?
- A deployment model: installation on the user’s/institute’s own server?
- Device-independent web-based access, also outside of your institute (e.g., via a VPN)?
- An exit strategy (outgoing migration)?
- Data migration from other systems (incoming migration)?

### Miscellaneous

Does the ELN offer:

- A classroom edition for teaching?
- Default language/multi-language support?

The following aspects are important to you:

- …
- …
- …
